# Transition to Motherhood: Adverse Childhood Experiences, and Support from Partner, Family and Friends

**DOI:** 10.1007/s10995-024-03922-6

**Published:** 2024-03-20

**Authors:** J. J. P. Mathijssen, E. Dirks, H. J. A. van Bakel

**Affiliations:** 1 TRANZO Department, Academic Collaborative Centre Youth, Tilburg University, Post Office Box 90153, 5000 LE Tilburg, The Netherlands; 2 Dutch Foundation for the Deaf and Hard of Hearing Child (NSDSK), 1073 GX Amsterdam, The Netherlands

**Keywords:** Adverse childhood experiences, Social support, Transition to parenthood, Motherhood constellation

## Abstract

**Background:**

The complex identity changes women have to go through to become mothers makes it a challenging transitional period. Especially, mothers who have experienced childhood adversity (ACEs) may be vulnerable to poor adjustment to motherhood. However, support from a partner, family and friends can act as a buffer to cope with this challenging transitional period. Therefore, the aim was to study whether ACEs and experienced social support (partners, family, and friends) were related to the adjustment to motherhood or ‘motherhood constellation’ of women after the birth of their first child.

**Method:**

Data were collected via an online questionnaire among first-time mothers from June–September 2020. Motherhood constellation was measured with four items based on the descriptions by (Stern, 1995) about the motherhood constellation, i.e. worries about Life/Growth, Emotional Engagement, Support Systems, Identity Organisation. Multiple regression analyses with pairwise deletion were conducted.

**Results:**

ACEs were related to all four themes of motherhood constellation, indicating that the more frequent these adverse experiences occurred in the past the more concerns, both about the child and herself, the mother had. Moreover, after controlling for ACEs and other forms of support, only support from friends was related to the use of support systems and identity organisation. Finally, statistically significant interactions were found between ACES and support from friends with life/growth and between ACES and support from family with identity organisation. These interactions indicated that contrary to the expectation the positive association between mother’s ACEs and worries was stronger for mothers who experienced more support.

**Conclusion:**

The consequences of ACEs seemed to show up in the transition to motherhood, indicating that interventions targeting first-time mothers should address the motherhood constellation that may arise from earlier adverse life experiences. Moreover, especially support from friends seemed to be associated with less worries among mothers. Social support has no buffering effect for the negative consequences of ACEs on the themes of motherhood constellation. Further research is clearly needed to get more insight into these themes and to understand the meaning of different types of social support during the transition to motherhood.

## Significance

**What is already known on this subject?**

Adverse childhood experiences (ACEs) predict maladaptive parenting cognitions, and impaired parent–child relationships.

**What this study adds?**

ACEs are also associated with the adjustment to motherhood of women after the birth of their first child. The more frequent ACEs occurred in the past the more concerns, both about the child and herself, the mother had. Moreover, especially support from friends seemed to be associated with less worries among mothers. Social support has no buffering effect for the negative consequences of ACEs on the themes of motherhood constellation.

Becoming a mother is a major developmental milestone, in which expectations, memories, and wishes about motherhood are considered (McCarthy et al., 2021; Prinds et al., 2014). The complex identity changes a woman has to go through to become a mother makes it a challenging transitional period (Beeck et al., 2022; Laney et al., 2015). The theory of the transition to parenthood by Stern (1995, 2004) proposes that when a woman becomes a mother she creates a ‘motherhood constellation’, a new identity regarding the baby and herself as well as expectations, anxieties, and goals regarding this role. Mothers experience several concerns, as reflected in themes they are worried about, such as being able to (1) keep the baby alive and protected (life / growth), (2) emotionally engage with the baby (emotional engagement), (3) create necessary support systems (support systems) and (4) combine the identity of being a mother with identities of being a wife/daughter/friend/employee (identity organization).

Especially women who have a history of childhood adversity may have more worries concerning the themes of motherhood constellation and may be vulnerable to poor adjustment to parenthood. Several studies have demonstrated that negative childhood experiences predict maladaptive parenting cognitions, and impaired parent–child relationships (Christie et al., 2017; DiLillo & Damashek, 2003; Gibb, 2002; Madigan et al., 2007; Pasalich et al., 2016; Rijlaarsdam et al., 2014; van Vugt & Versteegh, 2020). From an intergenerational perspective this means that negative childhood experiences can lead to perpetuation in the next generation via maladjustment to parenthood (Cicchetti & Toth, 2016).

Ecological parenting models highlight the impact of social support (i.e., exchange of emotional, social, and tangible resources) on parenthood adjustment (Belsky, 1984). Social support can act as a buffer to cope with this challenging transitional period. Indeed, research has shown that social support is essential for enabling women to cope with the transition to motherhood (Habel et al., 2015; Hamelin-Brabant et al., 2015; Mbekenga et al., 2011; McLeish & Redshaw, 2015; Chantal Razurel & Kaiser, 2015; Ni & Siew Lin, 2011; Razurel et al., 2013; De Sousa Machado et al., 2020). Moreover, especially in the context of adverse childhood experiences (ACEs) social support is important to take into consideration, as social support can diminish the effects of these adverse experiences (Christie et al., 2017).Based on the stress-buffering theory it could be assumed that social support moderates the relation between ACEs and motherhood constellation (Lazarus & Folkman, 1984).

Common sources of support for first-time mothers are partners, family, and friends (Eslahi et al., 2021). Particularly support from the spouse seems to have impact on the adaptation to motherhood (Cox et al., 1989; Razurel et al., 2011). Instability or strain in this partner relationship can have a strong effect on parents’ ability to cope with the challenging task of caring and parenting (Lennon & Heaman, 2015). Other studies have suggested that grandmothers are an important source of support during the postnatal period (Darvill et al., 2010; Taubman–Ben-Ari, 2019). Although, also friend support is assumed to be related to the ease of transitioning to motherhood. (Ni & Siew Lin, 2011), the different sources of support that mothers may receive have rarely been investigated in one study.

Therefore, the aim of this study was to investigate whether ACEs and social support (partners, family, and friends) are related to the motherhood constellation of women after the birth of their first child. Moreover, the study tested whether the association between adverse childhood experiences and motherhood constellation varied for the different levels and different sources of support.

## Method

### Procedure & Participants

Participants were recruited through social media (Facebook and LinkedIn) and children’s health care centers in the Netherlands between June and September 2020. Parents (of children born between March and June 2020) were asked to fill out an online questionnaire. In total 1096 parents, both mothers and fathers, completed the questionnaire (96.7% mothers, n = 1060). Of these 1060 mothers 465 (43.9%) had given birth to their first child. More information of these mothers is given in Table 1. Generally, mothers reported few concerns, especially about keeping the baby alive and protected and about emotional engagement. Moreover, the mothers did not experience many childhood adversities. Finally, on average mothers felt very supported by their partners, family and friends.

**Table 1:** Background characteristics of first-time mothers (n = 465)

	N (%) Mean (SD)
Family structure	
Two parents with child	443 (95.3%)
One parent with child	18 (3.9%)
Other	4 (0.8%)
Gender child	
Boy	236 (50.8%)
Girl	229 (49.2%)
Premature*	26 (5.6%)
Age child (in weeks, 1–26)**	11.0 (4.8)
Age mother (16–47)	30.2 (4.0)
Education level***	
Lower	16 (3.4%)
Medium	137 (29.5%)
High	312 (67.1%)
Adverse childhood experiences (1–10)	2.2 (2.0)
Support partner (1–7)	6.4 (1.0)
Support family (1–7)	6.0 (1.0)
Support friends (1–7)	6.0 (0.9)
Life / growth concerns (1–7)	1.7 (1.2)
Emotional engagement (1–6)	1.4 (0.9)
Support systems (1–7)	2.0 (1.4)
Combining different identities (1–7)	3.0 (1.7)

* Born before the end of the full term of gestation, i.e., earlier than 37 weeks

** Between parentheses in the first column; the range of given answers

*** The lower education level includes primary education and prevocational secondary education. The medium education level includes upper secondary education and vocational training. Higher education refers to universities of applied sciences and research universities

The Ethics Review Board of Tilburg University [RP186] approved the study. Participants provided informed consent before completing the survey and had a chance to win a prize (worth of 40 euros) through a raffle.

## Measures

*Adverse childhood experiences* were measured with one single question “Did you experience adverse experiences in the family in which you grew up during your childhood (including humiliation, neglect, lack of attention, physical maltreatment)? The score ranged from 1 (never) to 10 (always/continually).

*Social support* was assessed with the Multidimensional Scale of Perceived Social Support (MPSS; (Zimet et al., 1988)). The MPSS, a 12-item self-report scale, has three subscales measuring perceived social support from partner (4 items e.g., “I have a partner in life who cares about my feelings”), family (4 items e.g., “My family really tries to help me”), and friends (4 items e.g., “I have friends with whom I can share my joys and sorrows”). Parents rated their agreement with each item on a seven-point Likert scale from 1 (strongly disagree) to 7 (very strongly agree). In the current study the total mean score for each source of support was utilized.

*Motherhood constellation* was measured with four items based on the descriptions by Stern (1995) about the motherhood constellation themes: (1) life / growth concerns “I am afraid I cannot protect my baby/keep my baby alive”; (2) emotional engagement “I am concerned whether I can love my baby unconditionally”; (3) support systems “I am concerned about being able to arrange and accept support (practical and emotional) in my neighborhood to fulfill my responsibilities as a parent”; (4) combining different identities “ I am concerned whether I can perform my other roles/ or identity that I have, in addition to parenthood”. Scores ranged from 1 (totally disagree) to 7 (totally agree.).

### Analyses

The data were analyzed with SPSS 28. 0 (bivariate Pearson correlations, multiple regression analyses). Four different multiple regression models, with pairwise deletion, were conducted to determine the relationship between ACEs, social support and the four themes of motherhood. All regression analyses controlled for age of the mother, age of the baby and prematurity. Moreover, interactions terms (ACEs *partner support, ACEs *family support, and ACEs*friend support) were included as covariates in the regression models. The ACEs and social support variables were centered (i.e. subtracting the mean from each case resulting in a new mean of zero) before conducting the regression analyses. All the independent variables were included in one block.

## Results

Table 2 shows the Pearson correlations between childhood experiences, current support from family, partner, friends, and the themes of the motherhood constellation. The associations (*r’*s) varied between − 0.03 (partner support and emotional engagement till -0.31 (friends support and support systems).

**Table 2:** Correlations between childhood experiences, support, and themes of the motherhood constellation (n = 465)

	ACE	Partner support	Family support	Friend’s support	Life/growth	Emotional engagement	Support systems
Partner support	− .12**						
Family support	− .35**	.26**					
Friend’s support	− .27**	.27**	.44**				
Life/growth	.17**	− .12*	− .06	− .11*			
Emotional engagement	.20**	− .03	− .11**	− .10*	.26**		
Support systems	.25**	− .11*	− .17**	− .31**	.32**	.39**	
Identity reorganization	.24**	− .11*	− .19**	− .21**	.34**	.25**	.42**

*p ≤ .05

**p < .01

The results of the multiple regression analyses are given in Table 3. ACEs are related to all four themes of the motherhood constellation, indicating that the more frequent these adverse experiences occurred in the mother’s past the more concerns, both about the child and herself as a mother, she currently has. Moreover, after controlling for ACEs and other forms of support, support from friends was related to the theme of support systems and the theme of combining different identities. The more support mothers perceived from friends the less worries and anxieties they had about creating support systems and combining different identities. Finally, statistically significant interactions were found between ACES and support from friends with life / growth and between ACES and support from family with identity. These interactions indicated that (1) the association between ACEs and worries about protecting the child was stronger for mothers who experienced more support from their friends and (2) the association between ACEs and worries about the combination of fulfilling different identities was stronger for mothers who experienced more support from their families.

**Table 3:** Multiple regression (Standardized Beta’s, n = 465)

	Life/growth	Emotional engagement	Support systems	Identity reorganization
Intercept	1.50	1.21	1.37	1.41
Age mother	.02	.05	.05	.11*
Age baby	.02	− .09	.03	.06
Premature	.02	.14**	.06	.03
ACE	.17**	.17**	.19**	.20**
Support Partner	− .09	.02	− .01	− .03
Support Family	.05	− .04	.001	− .07
Support Friends	− .10	− .06	− .27**	− .13*
ACE * Family	− .02	.08	.08	.14*
ACE * Partner	− .05	− .06	− .02	.04
ACE * Friends	.13*	.02	.02	− .06
Adjusted R^2^	.03	.05	.11	.09

## Discussion

The aim of this research was to study the associations between ACEs and (the moderating role of) support from partner, family, and friends, and mothers’ ‘motherhood constellation’.

ACEs were related to all four themes of the motherhood constellation, indicating that the more frequent adverse experiences occurred in the past, the more worries mothers had about the capability protecting the child, loving the child, managing, and accepting support and combining parenthood with other roles and identities. These associations were found after controlling for the age of the mother, age and gender of the baby, prematurity, and different sources of support. Moreover, only support from friends was, independently from the other sources of support, related to two themes of motherhood, more precisely, with the aspects referring to the view of self as a parent (i.e., (1) support systems: managing, and accepting support and (2) identity organization: combining parenthood with other roles and identities). Although partner, family, and friend support were statistically significant related to each other, our findings demonstrated that these different sources of support are clearly distinguishable.

To the best of our knowledge, the themes of the motherhood constellation were especially studied in groups of pregnant women (Innamorati et al., 2010) or in the first year of life of preterm babies (Bortolin & Donelli, 2019). Both studies found that the life/growth theme was the most salient theme. In our study mothers reported, compared to other themes, a higher level of worries with the ‘identity organization theme’ about the combination of fulfilling different roles. It seems indeed evident that pregnant women and mothers of preterm babies are more engaged in the life/growth theme and are more concerned about their child’s health, survival and growth. In our sample, with relatively few babies being preterm (6%), other worries seemed to be of interest. When survival of the infant is less of a concern, mothers might focus on the other social roles they perform with the associated anxieties they experience in these processes.

Our finding that ACEs are related to both worries about the child and the identity as a mother corresponds partly with the results of a scoping review of Christie et al. (2017). The reviewed studies showed that parents with a history of maltreatment had more negative views of their child. However, regarding the identity as a parent theme the studies reported both positive and negative experiences. For example, for some women, the role of being a mother was experienced as a source of a new beginning with a positive view of the future (Aparicio et al., 2015). On the other hand, in the study of Michl et al. (2015) it was found that mothers with a history of childhood maltreatment had a higher level of self-criticism with negative and critical reflections on their role as a parent. Since we did not ask mothers the way they experience their new role in the light of possible ACEs, it might be that they experience the transition to motherhood as a new beginning with the ability to parent their child differently than they had been raised. However, our results clearly demonstrated that mothers with ACEs report more motherhood concerns.

Strikingly, and contrary to what has been assumed, it was not the partner support which was most related to the motherhood constellation. Although, partner support has consistently been identified as a factor that, protect against maternal stress and lessen symptoms of depression and anxiety (Pilkington et al., 2016; Stein et al., 2014), when controlled for other sources of support, partner support was not related to worries of new mothers, anymore. Possibly, the low variance in partner support could have had an important impact on the statistical power of our analysis. However, support from family members and friends with children who already have experience with the uncertainties of parenthood and know how to integrate different roles might provide the additional support (e.g., advice) that new mothers need in this postpartum period beyond the emotional support they receive from their partners. This result is in accordance with a recent meta-analytic review by Kelley et al (2021) who found that particularly familial support (beyond partner support) is an essential component of balancing work and family.

After controlling for ACEs, and other sources of support, family support was not related to worries of new mothers, anymore. In other studies, particularly mothers of first-time mothers have been identified as a main source of support (Leahy-Warren et al., 2012; Taubman–Ben-Ari, 2019). However, in our study we only asked whether the mother felt supported by their family in general, and not specifically by her own mother. Only friend support seemed to be related to worries of the mother, particularly with the aspects referring to the view of herself as a parent. The lower the perceived support from friends the more worries mothers had about (1) managing and accepting support and (2) combining parenthood with other roles and identities. Actually, it is not surprising that friend support is related to these two themes. When mothers report to experience support from friends it seems obvious that she is able to accept support. On the other hand, the associations between perceived friend support and worries about the role and identity of the self as a mother were only small to moderate, indicating that they are distinguishable concepts. The study of Zhou & Taylor (2022) hypothesized that especially in the case of single mothers’ family and friend support are important. However, our study demonstrated that particularly friend support is important, even when partner support is available.

A striking result was the positive interaction effect between ACEs and family support with the challenging theme of combining parenthood with other roles and identities. John Bowlby (1982) hypothesized that individuals exposed to rejection or neglect by attachment figures are likely to view themselves as not deserving affection or support. Possibly, in case of receiving family support later in live makes them extra aware of their shortcomings in childhood and makes them question whether they can properly fulfill their role as a mother in combination with their other roles. Another explanation could be that social support can have both beneficial and negative effects (e.g., Coyne & DeLongis, 1986). One more unexpected finding was the positive interaction effect between ACEs and friend support with the motherhood theme worrying about the ability of keeping the baby alive and protected. This indicates that the combination of ACEs and higher friend support is related to more worries. Possibly, this friend support makes vulnerable mothers uncertain about their ability to take good care of their child's life and growth.

Social support was hypothesized to be a protective factor in the transition to motherhood. Based on the main-effect model, social support would have a general positive effect regardless of ACEs (Cohen & Wills, 1985). According to the buffering hypothesis, social support is assumed to be beneficial and protects individuals from possibly negative consequences of adverse experiences (Yap & Devilly, 2004). In our study, we did not find confirmation for either hypothesis. In general, mothers reported high levels of support from their partner, family, and friends (on average 6.0 or higher on a scale from 1 to 7). However, this does not mean that support in itself is sufficient to play a role in reducing maternal worries and anxieties. Moreover, researchers have also demonstrated that supportive relationships are not necessarily free from conflict (e.g.,Burger & Milardo, 1995; Pierce et al., 1991).

Although ACEs were clearly related to all four themes of the motherhood constellation, in general, the ACEs, social support and both child and mother characteristics only explained a small to medium proportion of the variance in the four themes of this motherhood constellation. Especially, the worries about the child could not properly explained by our models, indicating that probably other factors are more important in explaining these worries. However, it is also important to keep in mind that the variance of these two variables was quite low, making it difficult to identify possible explaining factors. On average, the mothers did not have many worries about ‘keeping their baby alive’ and ‘emotional engagement. Osman et al. (2010) showed that among first-time mothers especially breastfeeding issues and information about normal infant care were the most mentioned concerns in a study about postpartum support telephone hotline. Moreover, measuring the motherhood constellation by using a self-report Likert-scale may have resulted in missing nuances in these themes. Since the motherhood constellation has been described by Stern (1995) as a ‘new psychic organization that emerges in the mother with or even before the birth of a baby’ or a ‘representation’, measuring the real existence of small to moderate worries and fears might be better captured by interviews with discourses of the mother’s early experiences with her baby (Innamorati et al., 2010).

In spite of the interesting results, there are some limitations. First, since we only studied whether mothers had a history of unspecified childhood maltreatment, it was not possible to unravel which form of maltreatment had the most effect on the transition to parenthood. Second, we only studied new mothers. Possibly, the associations between ACEs, social support and worries about parenthood are different for mothers and fathers. Only 23 fathers of a first child filled out the questionnaire, refraining us to analyse those data. Third, the data are limited in that information regarding different supportive provisions (e.g., instrumental support, emotional support, financial support), different family ties (e.g., in-laws, parents, siblings) and different relationship dimensions (e.g., conflict, stress) were not obtained. Consequently, differential associations for different supportive provisions, family ties, and relationship dimensions could not be examined in the present study. Fourth, since the participants were recruited through social media and children’s health care centers the sample was not random. Therefore, the results are not generalizable to all first-time mothers. Moreover, it is important to keep in mind that our survey had an overrepresentation of highly educated mothers. Although the percentage of highly educated people is increasing in the Netherlands, in 2021 about 55% of the 25 till 35 years old was highly educated, which is less than the percentage in our survey. Fifth, we should be aware that measuring ACEs with self-report may result in recall bias. The review by Hardt and Rutter (2004) demonstrated that retrospective reports are likely to provide underestimates of the incidence of ACEs. Healthy adults tend to underreport, rather than that adults with problems or a disorder overreport ACEs. Nevertheless, we expect the use of retrospective recall in adult life to be valid enough to warrant its use in studies, even with some significant underreporting and likely some bias.

Future research would benefit from studies with more diverse samples including both pregnant women and women who have just given birth, mothers with a known history of ACEs, and lower educated mothers. Moreover, studying more specified ACEs makes it possible to unravel its distinguishing effect on the transition to motherhood. Finally, future studies should include information regarding different supportive provisions and different relationships dimensions from both partners, family and friends.

In conclusion, the results of this investigation demonstrate that it ACEs are, although weak, associated with the four themes of motherhood for first-time mothers. This indicates that interventions targeting first-time mothers should address the motherhood constellation that may arise from earlier adverse life experiences.Moreover, especially friend support is, independent from ACEs and other forms of social support, related to worries about the view of mothers of themselves as a parent. Finally, no buffering effect of social support for ACEs on the four themes of motherhood was found. On the contrary, social support even seems to have a negative effect in some cases. Future research is clearly needed to get more insight into the themes of motherhood constellation and to understand the meaning of the different types of support during the transition to motherhood.

## Data Availability

Not applicable.
